# Supplementary material for: Prebiotic chiral transfer from self-aminoacylating ribozymes may favor either handedness
Source: Nat Commun. 2024 Sep 12;15:7980. doi: 10.1038/s41467-024-52362-x (PMC11393417; doi:10.1038/s41467-024-52362-x)
Supplement: Supplementary file 1 — Supplementary Information [file 41467_2024_52362_MOESM1_ESM.pdf]

Supplementary Information

Supplementary Note 1

Determination of enantioselectivity index from the integrated rate law

The concentration of D-BFO ($[D]$) and L-BFO ($[L]$) are assumed to be equal and constant, since BFO is synthesized in a racemic mixture and [BFO] >> [RNA]. At time $t = 0$, let the relative concentration of unmodified RNA ($[R]$) be equal to 1, and the concentration of aminoacylated RNA is 0. Aminoacylated RNA may be modified by D-BFO, giving product *RD*, or by L-BFO, giving product *LD*. The concentration constraints are:

(1) $$[L] = [D] \equiv B$$

(2) $$[R] + [RL] + [RD] = 1$$

The chemical system consists of two irreversible reactions:

(3) $$R + L \xrightarrow{k_L} RL$$

(4) $$R + D \xrightarrow{k_D} RD$$

where k_L and k_D are the rate constants for the reaction with each respective stereoisomer. The change in [R] is given by the following differential equation:

(5) $$\frac{d[R]}{dt} = -k_L[R]B - k_D[R]B$$

the analytical solution to which is

(6) $$[R(t)] = Ae^{-B(k_L+k_D)t}$$

where *A* represents the maximum fraction of aminoacylated RNA. Focusing on one stereoisomer, the change in L-aminoacyl-RNA is given by the following differential equation:

(7) $$\frac{d[RL]}{dt} = [R]Bk_L$$

Substituting (6) for R gives

(8) $$\frac{d[RL]}{dt} = ABk_Le^{-B(k_L+k_D)t}$$

the analytical solution to which, given the initial conditions, is

(9) $$[RL] = \frac{Ak_L}{k_L+k_D}(1 - e^{-B(k_L+k_D)t})$$

Similarly for D-aminoacyl-RNA,

(10) $$[RD] = \frac{Ak_D}{k_L+k_D}(1 - e^{-B(k_L+k_D)t})$$

Note that $k_D + k_L$ can be estimated from fitting of [RL]+[RD] vs. time to a pseudo-first-order rate equation. Note that the ratio k_D/k_L can be estimated from the ratio of amplitudes upon fitting [RL] and [RD] to equations 9 and 10, respectively, given data from multiple [BFO], or from direct calculation given a single [BFO], and that the ratio [RD]/[RL] = k_D/k_L is independent of time.

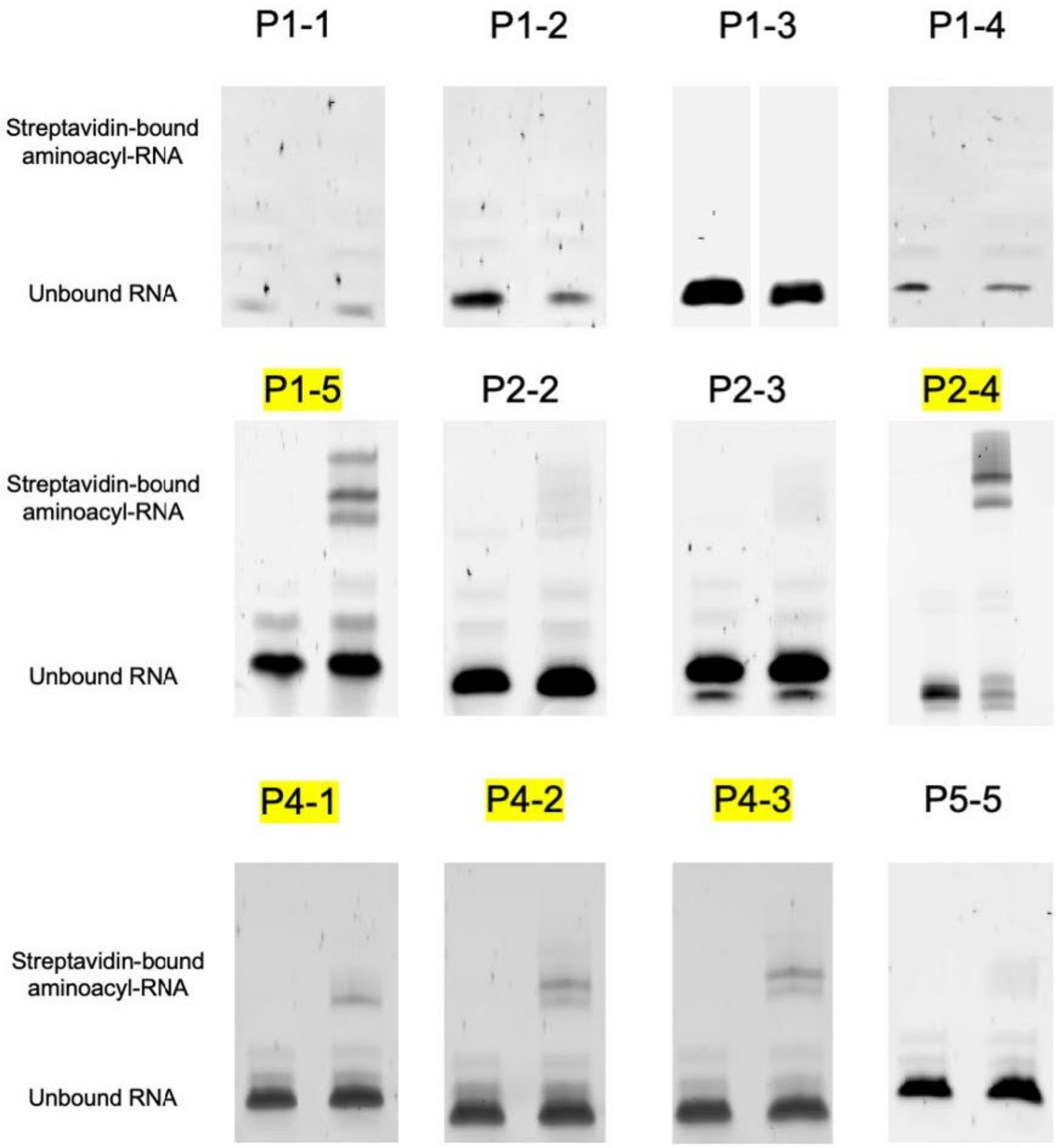

Supplementary Figure 1. Electrophoretic mobility shift assays (EMSA) of aminoacyl-RNA with streptavidin before (-BFO, left lane) and after (+BFO, right lane) incubation with BFO for 12 candidate ribozyme sequences. Based on these results, the five sequences with the greatest amount of reaction product were chosen for further analysis (highlighted yellow). For ribozymes that were not chosen for further analysis, the data shown is the only experimental replicate. For ribozymes that were chosen, the results shown are representative of at least triplicate independent experiments.

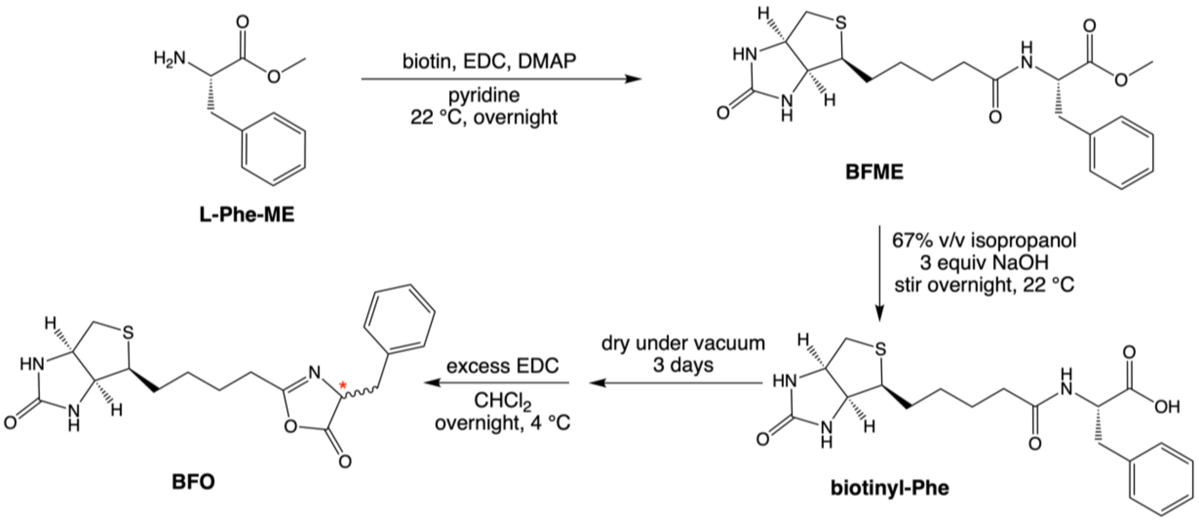

Supplementary Figure 2. Biotinyl-Phe-5(4*H*)-oxazolone (BFO) synthesis method (see Methods). The chiral center corresponding to the α-carbon is indicated with a red asterisk. ME = methyl ester.

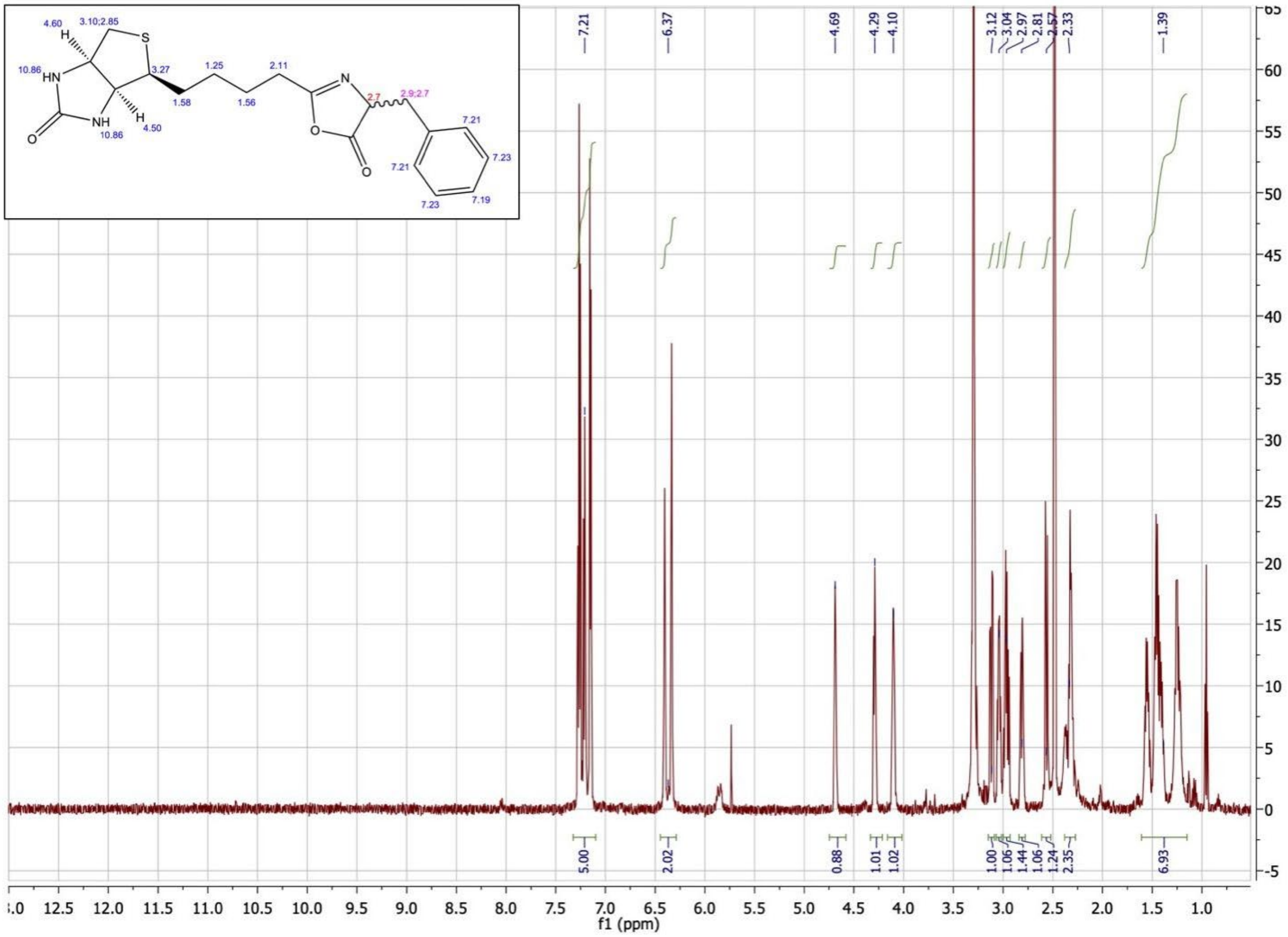

Supplementary Figure 3. Proton nuclear magnetic resonance (H[1] NMR) spectrum of BFO in deuterated DMSO. Peak centers labeled at top. Integrated values shown at bottom. Inset: structure of BFO with predicted H[1] NMR shifts.

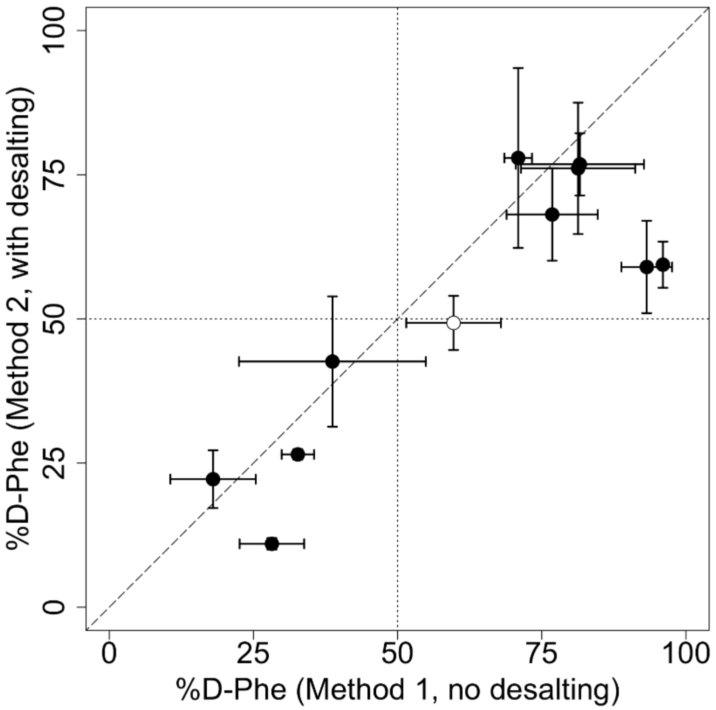

Supplementary Figure 4. Verification of racemic Phe from BFO and validation of chiral chromatography procedure. HPLC-MS data used for enantioselectivity analyses were obtained by method 1 (6 M HCl, no desalting or derivatization) and validated by method 2 (acid vapor hydrolysis, desalting column, and derivatized by *o*-phthaldialdehyde/*N*-acetyl-L-cysteine). Each point represents the mean of experimental triplicates. Error bars indicate standard error of the mean. The negative control (open circle) shows the result of 500 nM BFO incubated overnight in 60 mM $Na_2B_4O_7$ (pH 9.5). Ribozymes were incubated with 1 mM BFO (closed circles). The identity line is shown as a dashed line. The slope of the straight line of best fit (0.725; R^2= 0.78) was not significantly different from 1 (p-value = 0.086; two-sided bootstrap test, 1000 replicates).

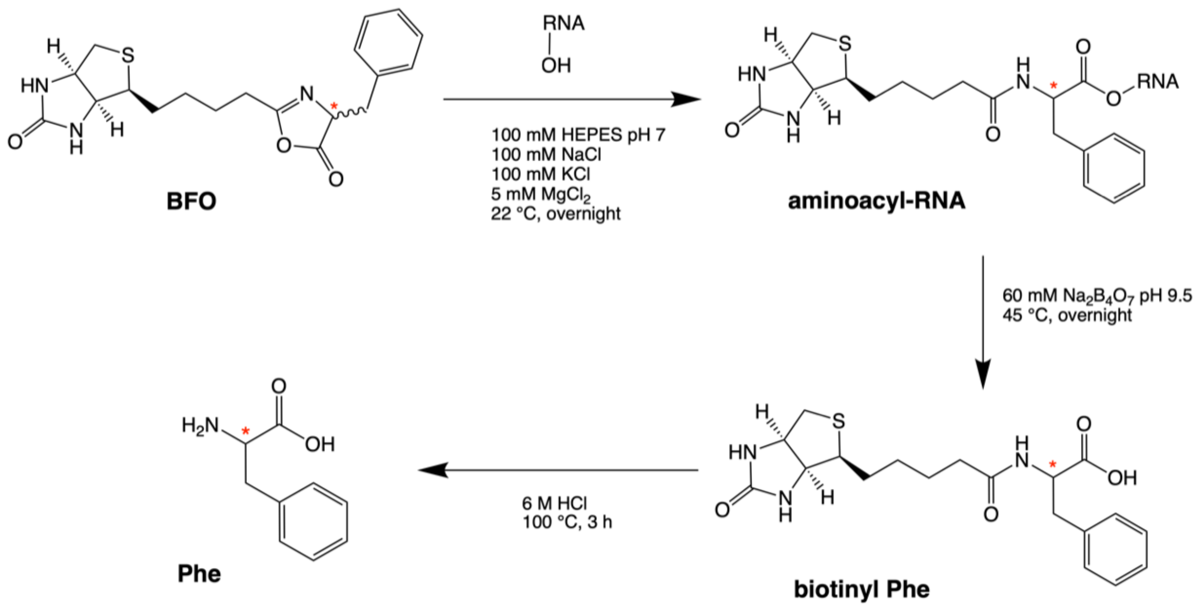

Supplementary Figure 5. Preparation of Phe for chiral separation using chiral chromatography. The chiral center for analysis is indicated with a red asterisk.

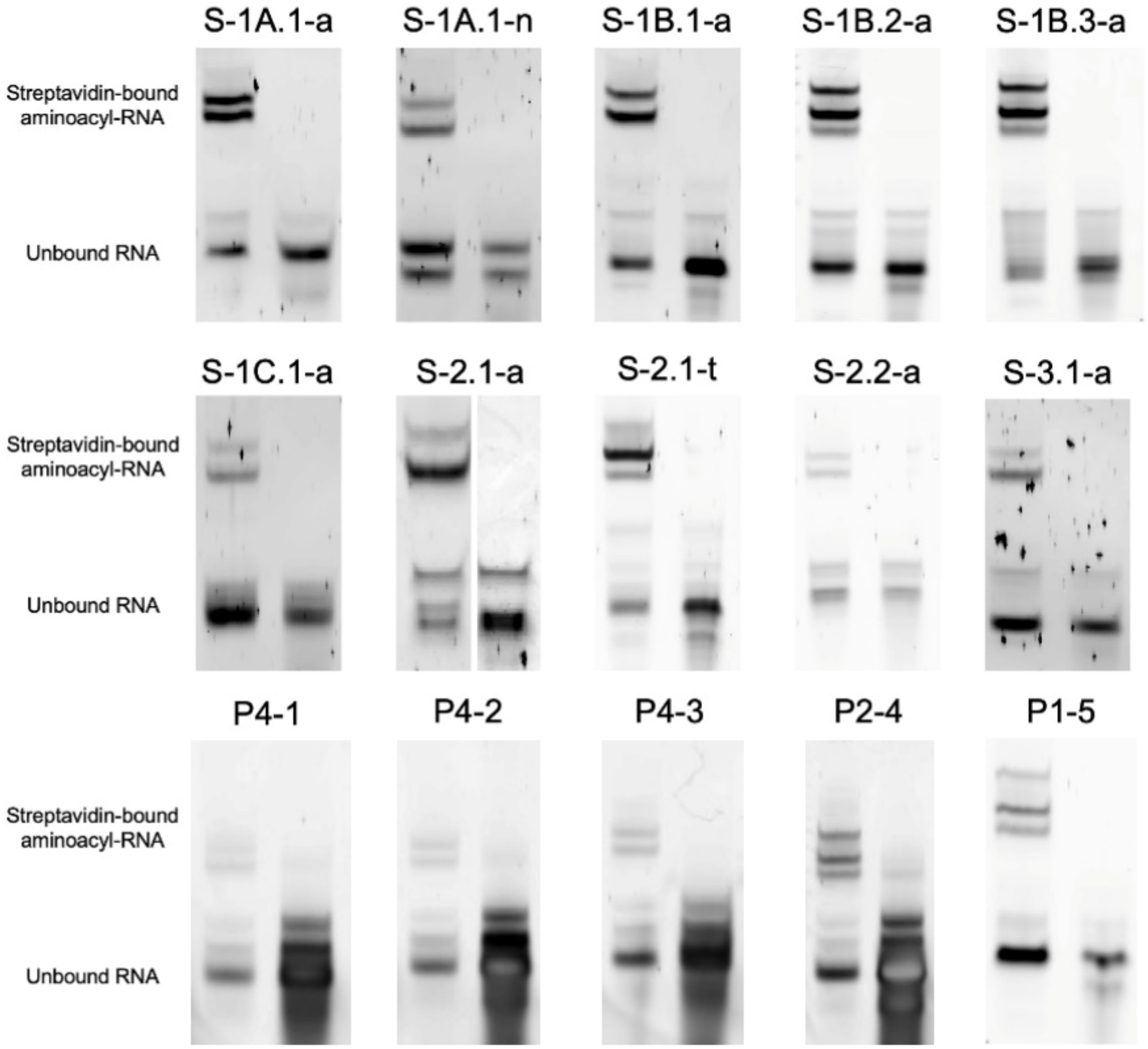

Supplementary Figure 6. Aminoacyl-RNA products and hydrolysis to release biotinyl-Phe for 10 previously identified ribozymes reacted with BFO. Electrophoretic mobility shift assays (EMSA) show self-aminoacylated ribozymes, incubated with streptavidin, before (left lane) and after (right lane) selective hydrolysis of the aminoacyl ester bond. As noted in earlier work (1), multiple bands were observed on the native gels in some cases; these may be caused by the presence of multiple RNA conformers or streptavidin oligomers. Results shown are representative of at least two independent replicate experiments for each ribozyme. Note: Ribozyme S-1A.1-a is also shown in main Figure 1A.

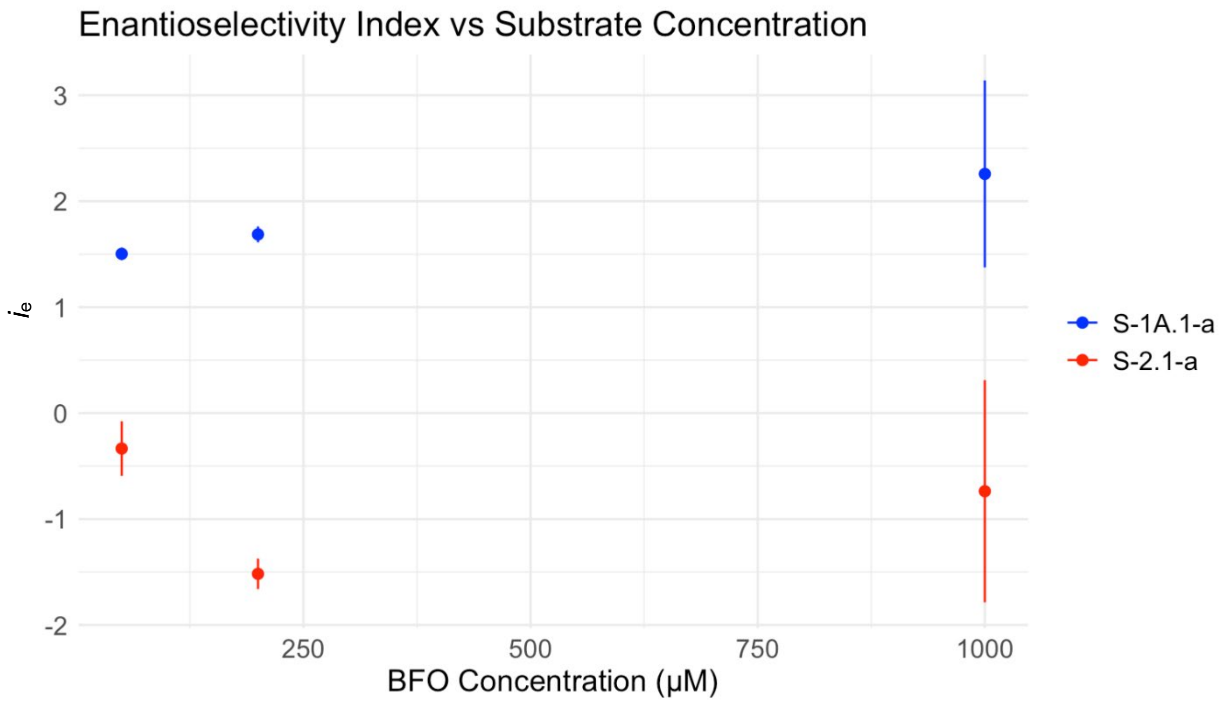

Supplementary Figure 7. Calculated enantioselectivity index (i_e) versus substrate concentration. A series of BFO concentrations was tested for ribozyme S-1A.1-a (blue) and S-2.1-a (red). i_e was calculated separately at each substrate concentration. Error bars represent standard deviation. Points indicate the mean and error bars represent standard deviation of n=3 independent experiments. See Supplementary Data for Source Data file.

Supplementary Reference

1. Janzen, E., Y. Shen, A. Vazquez-Salazar, Z. Liu, C. Blanco, J. Kenchel, and I. A. Chen. 2022. Emergent properties as by-products of prebiotic evolution of aminoacylation ribozymes. *Nat Commun*. 13(1):3631.

Source Data for Supplementary Figures 1 and 6

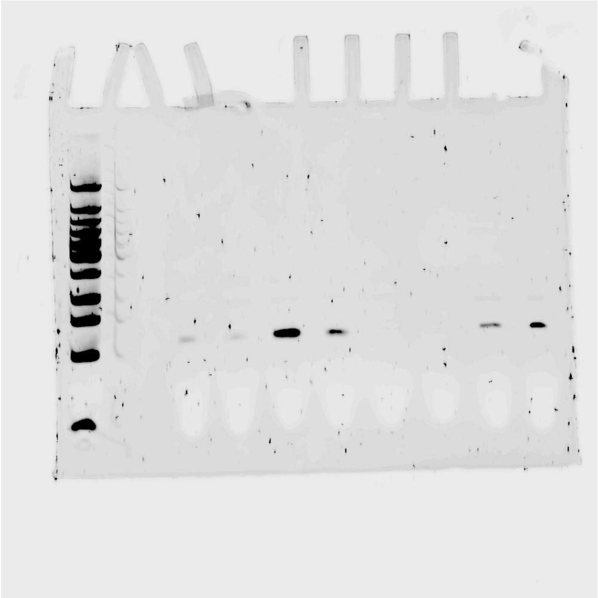	Supplementary Figure 1. From left to right: MWM, empty, (-BFO/+BFO) for P1-1, P1-2, two empty lanes, and (+BFO/-BFO) for P1-4. Note that in Supplementary Figure 1, the image for P1-4 is flipped relative to the source image. Flipping the image is equivalent to physically flipping the gel, and does not alter the lane identity.
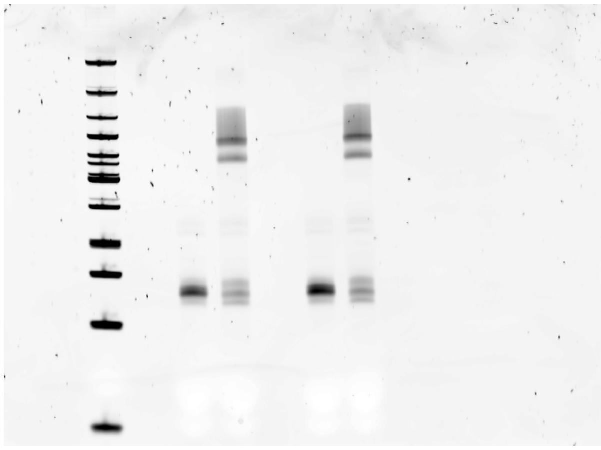	Supplementary Figure 1 and Fig 1. MWM, -/+ BFO: P2-4 (two replicates; second replicate shown in manuscript)
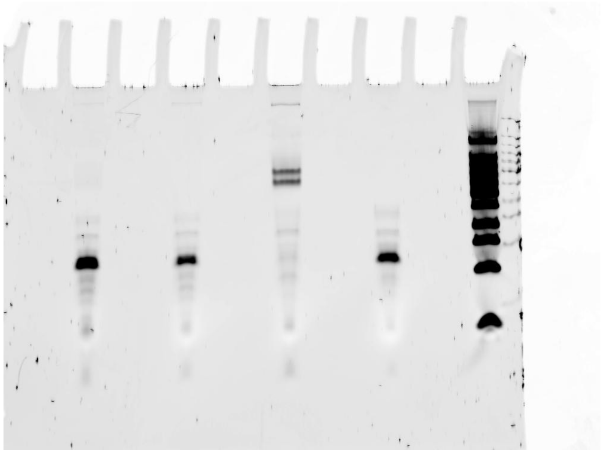	Supplementary Figure 1. P1-3, lane 2 = -BFO, lane 4 = +BFO. Other lanes not used in manuscript.

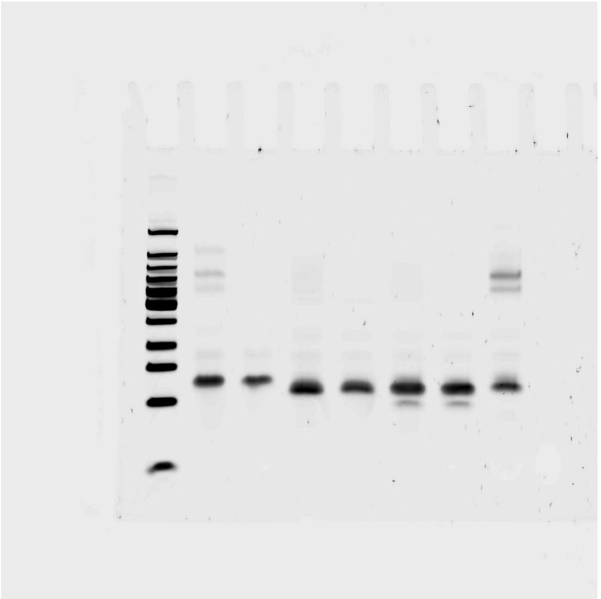	Supplementary Figure 1. From left to right: MWM, +BFO/-BFO: P1-5, P2-2, P2-3. (Last lane not used in manuscript) Note that in Supplementary Figure 1, these images are flipped relative to the source image. Flipping the image is equivalent to physically flipping the gel, and does not alter the lane identity.
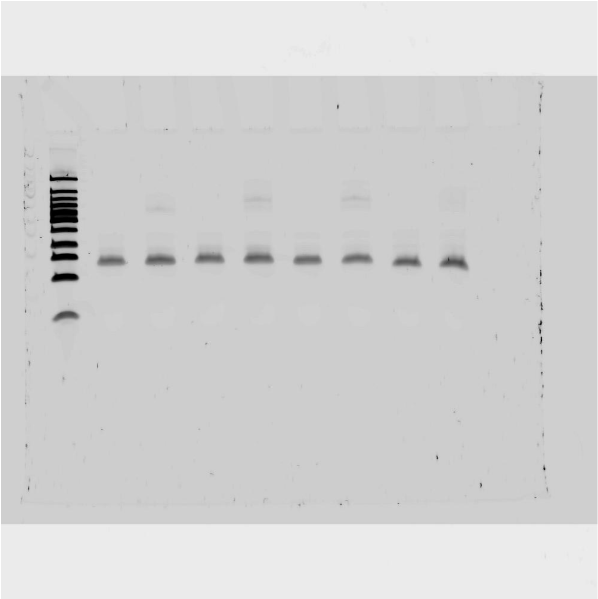	Supplementary Figure 1. From left to right: MWM, -/+BFO: P4-1, P4-2, P4-3, P5-5.
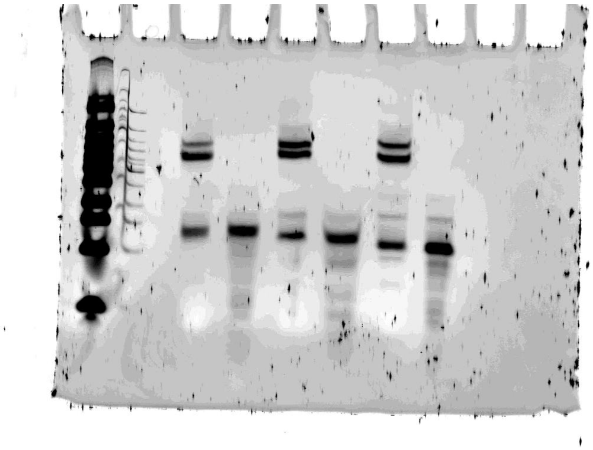	Supplementary Figure 6 (and Figure 1A). From left to right: MWM, empty lane, pre/post hydrolysis: S-2.1-t (not used in main manuscript), S-1A.1-a, S-1B.1-a

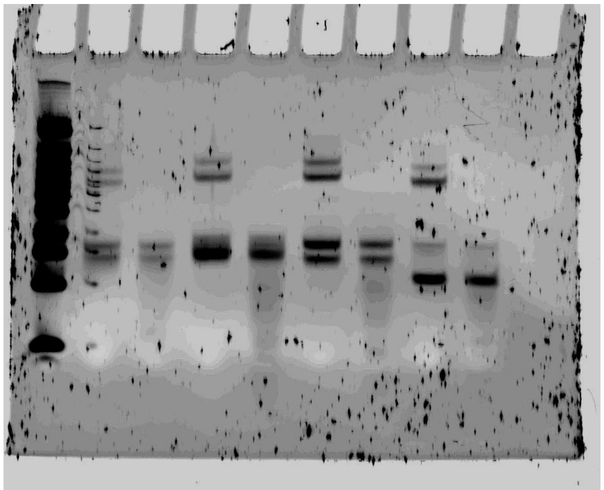	Supplementary Figure 6. From left to right: MWM, pre/post hydrolysis: S-2.2-A (not used in manuscript), S-1C.1-a, S-1A.1-n, S-3.1-a
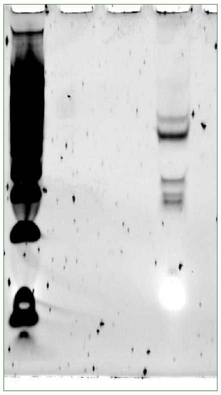	Supplementary Figure 6. MWM, two empty lanes, and S-2.1-a pre hydrolysis
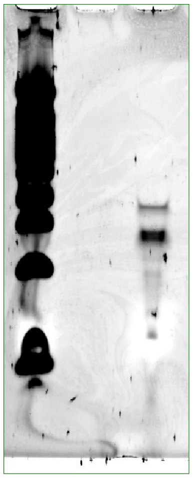	Supplementary Figure 6. MWM, empty lane, and S-2.1-a post hydrolysis

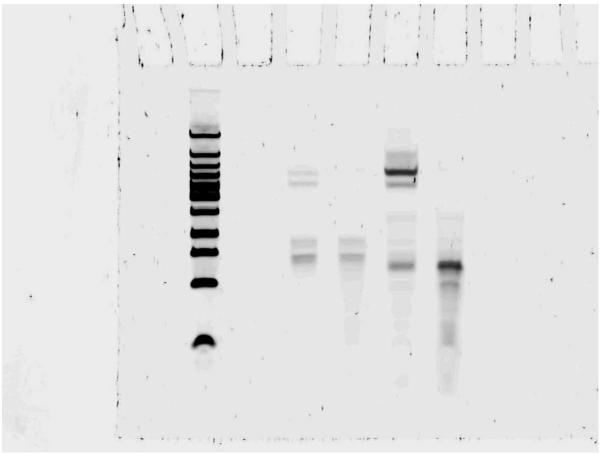	Supplementary Figure 6. Left to right: MWM, empty lane, pre/post hydrolysis for S-2.2-a and S-2.1-t
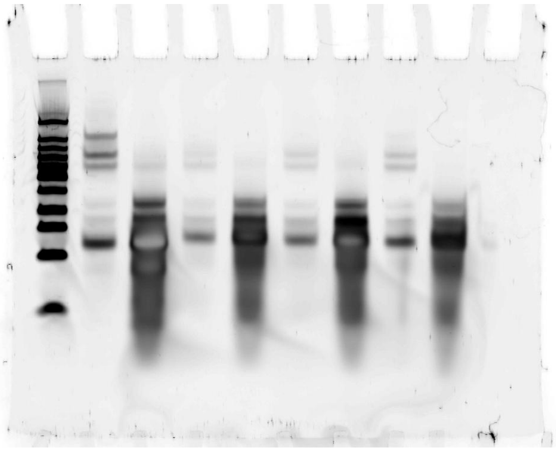	Supplementary Figure 6. Left to right: MWM, pre/post hydrolysis: P2-4, P4-1, P4-2, P4-3.
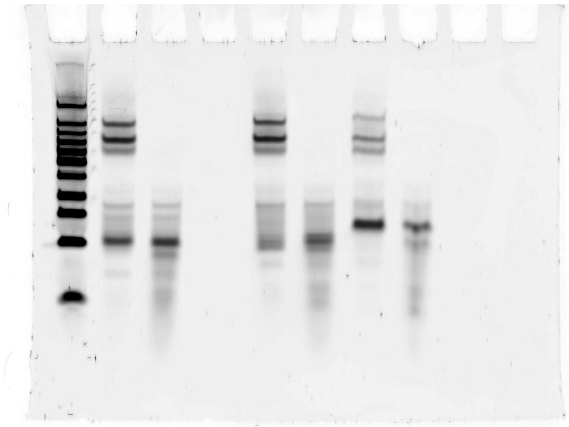	Supplementary Figure 6. From left to right, pre/post hydrolysis: MWM, S-1B.2-a, empty lane, S-1B.3-a, P1-5
